# Genome-Wide Association Studies Detect Multiple QTLs for Productivity in Mesoamerican Diversity Panel of Common Bean Under Drought Stress

**DOI:** 10.3389/fpls.2020.574674

**Published:** 2020-11-12

**Authors:** Paula Arielle Mendes Ribeiro Valdisser, Bárbara S. F. Müller, Janeo Eustáquio de Almeida Filho, Odilon Peixoto Morais Júnior, Cléber Morais Guimarães, Tereza C. O. Borba, Isabela Pavanelli de Souza, Maria Imaculada Zucchi, Leandro G. Neves, Alexandre S. G. Coelho, Claudio Brondani, Rosana Pereira Vianello

**Affiliations:** ^1^Biotechnology Laboratory, EMBRAPA Arroz e Feijão, Santo Antônio de Goiás, Brazil; ^2^Genetics and Molecular Biology Graduate Program, Institute of Biology, UNICAMP, Campinas, Brazil; ^3^Department of Horticultural Sciences, University of Florida, Gainesville, FL, United States; ^4^Bayer Brazil – Crop Science, Coxilha, Brazil; ^5^Department of Genetics and Plant Breeding, School of Agronomy, Federal University of Goiás, Goiânia, Brazil; ^6^Plant Physiology Laboratory, EMBRAPA Arroz e Feijão, Santo Antônio de Goiás, Brazil; ^7^Postgraduate Program in Biological Sciences, Institute of Biological Sciences, Federal University of Goiás, Goiânia, Brazil; ^8^Agribusiness Technology Agency of São Paulo State, Agriculture and Food Supply Secretary of São Paulo, Piracicaba, Brazil; ^9^Rapid Genomics, Gainesville, FL, United States; ^10^School of Agronomy, Federal University of Goiás, Goiânia, Brazil

**Keywords:** *Phaseolus vulgaris*, DArTseq markers, CaptureSeq, genetic diversity, seed-weight, yield, GWAS, candidate markers

## Abstract

Drought stress is an important abiotic factor limiting common bean yield, with great impact on the production worldwide. Understanding the genetic basis regulating beans’ yield and seed weight (SW) is a fundamental prerequisite for the development of superior cultivars. The main objectives of this work were to conduct genome-wide marker discovery by genotyping a Mesoamerican panel of common bean germplasm, containing cultivated and landrace accessions of broad origin, followed by the identification of genomic regions associated with productivity under two water regimes using different genome-wide association study (GWAS) approaches. A total of 11,870 markers were genotyped for the 339 genotypes, of which 3,213 were SilicoDArT and 8,657 SNPs derived from DArT and CaptureSeq. The estimated linkage disequilibrium extension, corrected for structure and relatedness (*r*^2^_*sv*_), was 98.63 and 124.18 kb for landraces and breeding lines, respectively. Germplasm was structured into landraces and lines/cultivars. We carried out GWASs for 100-SW and yield in field environments with and without water stress for 3 consecutive years, using single-, segment-, and gene-based models. Higher number of associations at high stringency was identified for the SW trait under irrigation, totaling ∼185 QTLs for both single- and segment-based, whereas gene-based GWASs showed ∼220 genomic regions containing ∼650 genes. For SW under drought, 18 QTLs were identified for single- and segment-based and 35 genes by gene-based GWASs. For yield, under irrigation, 25 associations were identified, whereas under drought the total was 10 using both approaches. In addition to the consistent associations detected across experiments, these GWAS approaches provided important complementary QTL information (∼221 QTLs; 650 genes; *r*^2^ from 0.01% to 32%). Several QTLs were mined within or near candidate genes playing significant role in productivity, providing better understanding of the genetic mechanisms underlying these traits and making available molecular tools to be used in marker-assisted breeding. The findings also allowed the identification of genetic material (germplasm) with better yield performance under drought, promising to a common bean breeding program. Finally, the availability of this highly diverse Mesoamerican panel is of great scientific value for the analysis of any relevant traits in common bean.

## Introduction

Prolonged drought episodes are critical to plant development and can have devastating impacts on crop productivity worldwide (Eisenstein, 2013; Madadgar et al., 2017). Agricultural losses due to drought are associated with both reduced crop areas and production losses, and studying these issues in an attempt to understand their impacts and consequences has become a major challenge for the scientific community (Lesk et al., 2016). In this context, major global changes are taking place in agriculture, such as the development of systems and practices that are more resilient to the impacts of climate change, with less vulnerability and greater adaptability, and the development of agricultural activities with lower greenhouse gas emission, more suitable production levels, improved distribution, less waste, and equitable access, among others (Food and Agriculture Organization [FAO], 2018). Developing countries are the most vulnerable to drought risks and have suffered approximately 80% of the damage caused by this stress over the 2006–2016 period (Food and Agriculture Organization [FAO], 2018). In the specific case of common bean, drought has affected its cultivation in several regions of the world, causing high production losses (Rao, 2001; Heinemann et al., 2016).

Among legumes, common bean (*Phaseolus vulgaris* L.) is a food of high nutritional value and of great economic and social importance. It is a valuable source of carbohydrates, proteins, and minerals, in addition to being rich in bioactive agents with a variety of human health benefits, including biological activities such as antioxidant, anti-inflammatory, antidiabetic, antihypertensive, and anticancer activities (Heredia-Rodríguez et al., 2016). Beans are part of the diet of more than half a billion people in Latin America and Africa, supplying up to 15% of total daily calories and 36% of total daily protein (Schmutz et al., 2014); they are grown in 126 countries, with an annual planted area of approximately 30.6 million hectares (Food and Agriculture Organization [FAO], 2016). Approximately 15% of these areas are in regions with severe drought, such as in Brazil, on the Peruvian coast, in northern Mexico and in dry regions of Africa (Singh, 2005). For common bean, diseases represent the major cause of productivity loss, followed by water stress (WS) (Singh, 1995), which affects approximately 60% of the crop areas and is the result of both drought periods during the crop cycle and irregular rainfall (Graham and Ranalli, 1997; McClean et al., 2011).

Drought tolerance is achieved by many traits, most of them with complex inheritance and low heritability, and all of these traits are related with grain yield (GY) in crops (Schneider et al., 1997b; Blair et al., 2012). In practical terms, selecting lines with higher yield potential under drought conditions is complicated because the extent of the effect depends on its duration and the intensity of WS and may be potentiated by other factors such as low fertility and soil acidity, diseases, and high temperatures (Blum, 2011). In addition, plants use different adaptive strategies for drought stress, such as escape or activation of tolerance and recovery mechanisms (Levitt, 1972). In common bean, several traits associated with drought tolerance have already been identified, including (a) more developed root systems (Sponchiado et al., 1989; Frahm et al., 2004); (b) translocation and accumulation of biomass to the seeds (Ramirez-Vallejo and Kelly, 1998; Rao et al., 2004; Polania et al., 2016); (c) adjustment of phenological traits, such as days to flowering and photosynthetic water-use efficiency, growth and development (Acosta-Gallegos and White, 1995; Rosales-Serna et al., 2004); and (d) the adjustment of physiological mechanisms associated with stomatal conductance, leaf area, and osmotic adjustment (Beebe et al., 2013; Lanna et al., 2016, 2018).

Nowadays, more efficient breeding strategies to release new cultivars have been demanded, and molecular tools could support that, based on accumulated knowledge. In most studies, genetic maps of biparental populations have provided the basis for quantitative trait loci (QTLs) identification for drought tolerance in common bean (Schneider et al., 1997a; Blair et al., 2012; Mukeshimana et al., 2014; Trapp et al., 2015; Villordo-Pineda et al., 2015; Briñez et al., 2017). The use of single-nucleotide polymorphism (SNP) markers, with a genome-wide distribution, has increased the power to identify markers in linkage disequilibrium (LD) with QTL/gene of interest. Different SNP genotyping approaches are currently available, such as restriction site–associated DNA sequencing (Willing et al., 2011), Genotyping-by-sequencing (Elshire et al., 2011) and diversity arrays technology (DArTseq) (Cruz et al., 2013). Such methods based on reduction of genome complexity requires a refined selection of the restriction enzymes in order to identify and genotype larger numbers of variants with a broad genome coverage, as shown for common beans by Ariani et al. (2016). The DArTseq methodology differs from the others by using two restriction enzymes, a common-cutting enzyme and a methylation-sensitive rare-cutting enzyme, preferentially fragmenting hypomethylated regions that are generally enriched with genes (Rabinowicz et al., 2005). In addition, the search for SNPs in specific genomic regions using the targeted sequence capture (CaptureSeq) methodology allowed the simultaneous identification of thousands of SNPs in regions of interest, allowing a genotyping refinement (Neves et al., 2013).

Increasing access to several high-throughput genotyping technologies has allowed for a large volume of studies using association mapping approaches. Several studies have reported single-SNP genome-wide association studies (GWASs) in common bean being evaluated in structured populations within the Andean and Mesoamerican gene pools. This sampling strategy by germplasm origin reduces genetic structure and extent of LD providing more resolution for discovering potentially useful causal variants. Using a panel of Mesoamerican accessions and high-density SNPs, Schmutz et al. (2014) and Moghaddam et al. (2016) have identified associations for several agronomic traits that affect common bean production, such as days to flowering and maturity, growth habit, lodging, and seed weight (SW), among others. Furthermore, Hoyos-Villegas et al. (2017) reported 27 associations for agronomic-related traits evaluated under drought and irrigated conditions in a Middle American diversity panel comprising 96 common bean genotypes. Recently, Andean and Mesoamerican diversity panels were developed and used to map production traits in both heat and drought stress environments (Oladzad et al., 2019). Based on Andean germplasm, genomic regions associated to cooking time (Cichy et al., 2015), biological nitrogen fixation (Kamfwa et al., 2015), and resistance to anthracnose (Zuiderveen et al., 2016) and to bacterial blight (Tock et al., 2017) have been identified by single-model GWASs. Other GWAS approaches that explore the aggregated effects of multiple SNPs in genomic segments, such as regional heritability mapping (Nagamine et al., 2012), increased the power to capture genomic regions associated with lodging, architecture, and yield in common beans (Resende et al., 2018). In addition, GWAS approaches using the genetic information provided by SNPs in a gene (gene-based GWASs) or SNPs in segments (segment-based GWASs, Bakshi et al., 2016) have been used as supplementary methods for SNP-based GWASs (Peng et al., 2010). As these methods explore the aggregated effects of several SNPs, including those with rare and low-frequency alleles, in genomic and gene segments, they have the potential to capture more of the genetic effects accounted for the traits, rather than the methods based on individual SNPs, in which isolated effects that contribute little to variance are not detected by GWASs (Resende et al., 2017).

The objectives of this study were (1) to identify SNPs with high representativeness over the Mesoamerican gene pool that are part of the Brazilian core collection, through DArTseq and CaptureSeq approaches; (2) to estimate the genetic parameters and the genetic structure and investigate genome-wide LD in cultivar and landrace germplasms; and (3) to identify genomic regions associated with 100-SW and yield under different water regimes and years of experimentation, using GWAS approaches based on single-SNPs and models that explore the combined effects of multiple SNPs using gene- and segment-based methods.

## Materials and Methods

### Plant Material

A total of 339 Mesoamerican common bean accessions, including 224 landraces from Brazil and 115 Brazilian and international cultivars/lines, were used. These genotypes are maintained as part of Embrapa’s Brazilian common bean core collection (CONFE), which is backed up in the Svalbard Global Seed Vault (Longyearbyen, Norway) (Supplementary Table 1). The accessions were multiplied in a greenhouse, ensuring homogeneity for genetic analysis, and individually collected for molecular analysis. Total genomic DNA was obtained using the Invisorb Spin Plant Mini Kit (Stratec Molecular, Berlin, Germany), followed by shipment to DArTseq (DArT Pty Ltd., Bruce, Australia) and RAPiD Genomics (Gainesville, FL, United States) facilities. The georeferenced landraces were plotted using the R package ggplot2 (Wickham, 2009) and the map provided through the *rnaturalearth* package (South, 2017).

### Phenotyping

The field experiments were carried out at the phenotyping site for drought tolerance located at the EMATER Experimental Station (Porangatu, Brazil) using two irrigation conditions, adequate water supply and imposing water restriction (WS). The experiments were conducted in 3 consecutive years (2014, 2015, and 2016), in the third season, or irrigated growing season (May–August), when rainfall is practically absent. The sample sizes are presented in Table 1.

**TABLE 1:** Estimates of the genetic parameters for grain yield (GY) and 100-seed weight (SW) traits in individual and joint phenotypic analyses conducted on field experiments with (WS) and without water restrictions (NS).

**Environment**	**NS-2014**		**WS-2014**		**NS-2015**		**NS-2016**		**WS-2016**		**NS joint analysis**		**WS joint analysis**	
**Traits**	**GY**	**SW**	**GY**	**SW**	**GY**	**SW**	**GY**	**SW**	**GY**	**SW**	**GY**	**SW**	**GY**	**SW**
**N**	533	537	533	539	298	298	253	253	252	254	531	531	527	528
**Avg**	2,391.00	26.81	517.40	20.60	1,187.00	22.24	1,947.00	26.77	798.80	21.31	1,870.00	27.78	705.20	23.23
**Minimum**	1,638.00	16.44	372.00	13.59	557.00	13.79	999.64	15.68	421.48	6.63	1,528.00	13.92	537.00	8.44
**Maximum**	4,025.00	56.80	986.00	42.67	2,148.00	48.06	3,130.00	50.67	1,575.00	37.99	2,371.00	51.51	973.00	40.58
**Access avg**	2,522.28	26.49	579.94	20.41	1,299.87	22.16	1,968.62	23.05	876.10	21.13	1,890.18	23.10	711.79	20.67
**Avg landraces**	2,546.89	26.02	584.02	20.15	1,302.41	21.50	1,945.19	22.14	880.96	20.88	1,892.37	22.57	713.53	20.45
**Avg lines/cult.**	2,467.76	27.54	570.92	21.00	1,295.62	23.28	2,005.51	24.48	868.00	21.54	1,885.74	24.17	708.21	21.11
*h*^2^	0.41	0.95	0.37	0.85	0.59	0.93	0.58	0.98	0.57	0.91	0.57	0.99	0.57	0.97
*r*_*gĝ*_	0.72	0.97	0.72	0.93	0.76	0.96	0.74	0.99	0.71	0.93	0.82	0.98	0.70	0.95
**CV (%)**	31.11	7.27	42.16	11.50	40.68	12.89	33.07	6.84	42.37	11.38	31.94	9.80	47.53	11.34

*N, no. of accesses evaluated; Avg, average; *h*^2^, heritability; *r*_*gĝ*_, genetic accuracy; CV, coefficient of variation; GY, grain yield kg ha^–1^; SW, 100-seed weight (grams).*

In 2014, 580 accessions from the Brazilian CONFE, composed of Andean and Mesoamerican accessions, were phenotyped in the field. The experiment was carried out using the Federer augmented block design, in which the plots consisted of three 3-m rows. The experiment comprised 20 blocks composed of 29 accessions and four controls each (BRS Estilo, BRS Esplendor, BRS Embaixador, and Jalo Precoce). In 2015 and 2016, the experiments were carried out using only the 339 Mesoamerican accessions, and the plant breeders decided to use the square lattice design with two replicates and four controls. Each experiment was composed of 18 blocks with 18 treatments. The blocks consisted of 3-m rows with spacing of 40 cm, with a density of 15–18 seeds per meter.

The water field treatments consisted of two watering regimes, irrigated [non-stress (NS)] and with WS. In the control, adequate condition of water in the soil was maintained throughout the cycle of plant development, in which water slides of approximately 25 mm were applied when the soil water potential at 0.15-m depth reached -0.035 MPa (Silveira and Stone, 1994). In the WS condition, the irrigation was maintained until the 20th day after seedlings emergence, followed by WS applying water slides of approximately 25 mm when the soil water potential at 0.15-m depth reached -0.070 MPa until the end of the cycle of the plants.

### Evaluation of Productivity Components

The components of productivity SW, expressed as weight per 100 grains (grams) and GY, obtained through the grain weight per plot transformed into kg ha^–1^ were evaluated. The components of variance and adjusted means for each genotype were obtained through individual analysis, by environment, and by joint analysis, involving the environments for each irrigation condition.

For the analysis conducted by year, considering the general model with repetition and block within repetition effects, the mixed linear model (MLM) described below was fitted:

$$y_{\text{ijkm}}=\,\mu +r_{j}+b_{k/j}+t_{m}+g_{i/m}+\varepsilon_{i\,j\,k\,m}$$

where *y*_*ijkm*_ is the observation of genotype *i* in repetition *j* in block *k* belonging to type *m*; μ is a constant; *r*_*j*_ is the random effect of repetition *j*; *b*_*k/j*_ is the random effect of block *k* within repetition *j*; *t*_*m*_ is the fixed effect of type *m* of genotypes, with one group of accession and one group of check cultivars; *g*_*i/m*_ is the effect of genotype *i* (accession, as random effect, or check cultivar, as fixed effect) within type *m*; and ε_*ijkm*_ is the experimental error associated with observation *ijkm*.

For the joint analysis of the 3 years combined, the MLM described below was fitted:

$$y_{i\,j\,k\,l\,m}=\mu +a_{l}+r_{j/l}+b_{k/j/l}+t_{m}+g_{i/m}+g\,a_{i\,l}+\varepsilon_{i\,j\,k\,l\,m}$$

where *y*_*ijklm*_ is the effect of genotype *i* in repetition *j* in block k in environment *l* belonging to type *m*; μ is a constant; *a*_*l*_ is the fixed effect of environment *l*; *r*_*j/l*_ is the random effect of repetition *j* within environment *l*; *b*_*k/j/l*_ is the random effect of block *k* within repetition *j* in environment *l*; *t*_*m*_ is the fixed effect of type *m* of genotypes, with one group of accession and one group of check cultivars; *g*_*i/m*_ is the effect of genotype *i* (accession, as random effect, or check cultivar, as fixed effect) within type *m*; *ga*_*il*_ is the interaction effect between the genotype *i* and the environment *l*; and ε_*ijklm*_ is the experimental error associated with observation *ijklm*.

The variance components were estimated by the restricted maximum likelihood (ML) method, according to Patterson and Thompson (1971), and because of the presence of unbalanced data set over the experiments (resulting from the loss of some experimental plots and different number of repetitions), the genetic values of each accession were predicted using the BLUP (best linear unbiased prediction) procedure, according to Henderson (1984). Based on the genetic, phenotypic, and residual variance components, the broad sense heritability (*h*^2^) was estimated at the level of accession means. The accuracy of selection was also estimated (*r*_*gĝ*_), corresponding to the square root of the heritability estimate, according to Resende and Duarte (2007).

The MLMs were analyzed using the lme4 package of the R platform (R Core Team, 2015). The BLUP-adjusted values for each genotype within each experiment and over experiments, obtained in joint analysis (BLUPj), were used for GWASs. Box plots were generated using software R v. 3.0.1 (R Core Team, 2015).

### DArTseq Genotyping

The DArTseq technology, proposed by Jaccoud et al. (2001), was developed by the company Diversity Arrays Technology Pty Ltd. (Bruce, Australia). After preparation of the samples, the DNA was sent to DArT P/L for genotyping. The markers were developed as described by Sánchez-Sevilla et al. (2015). The method was based on reducing the complexity of the genome using the *Pst*I*–Mse*I restriction enzymes. The DArTseq data were used to generate the dominant SilicoDArTs (presence or absence of the hybridized fragment) and SNP markers present in the genomic representations. The sequences were processed, followed by SNP and SilicoDArT calling using DArT Pty Ltd.’s proprietary pipelines. The parameters “reproducibility” (percentage of technical replicate pairs scoring identically for a given marker) and “call-rate” (percentage of samples for which a marker was scored) were used to determine the quality of markers (Kilian et al., 2012).

### CaptureSeq Genotyping

A set of 5,293 differentially expressed genes under drought stress (Pereira et al., 2020) were initially used as target regions of the genome. The target capture (CaptureSeq) methodology was carried out by the company RAPiD Genomics (Gainesville, FL, United States). To target the sequences, the segments were BLASTed against the *P. vulgaris* reference genome (“Pvulgaris_218_v2.0.fasta”) (Schmutz et al., 2014). The resulting sequences were subjected to RAPiD Genomics probe design and synthesis. A set of 175 accessions, part of the 339 accessions used for the DArTseq analysis, was processed and hybridized to custom probes followed by next-generation sequencing (NGS) using HiSeq 2000 (Illumina) as described by Neves et al. (2013). Bioinformatics filtering steps were applied to obtain high-quality SNP data across 5,050 genes, and only one probe per gene was considered as callable region for on-target SNP discovery and identification.

### Imputation

Imputation of the SNPs was performed using the software NGSEP v. 3.0.1 (Duitama et al., 2014), suitable for endogamous populations, using the parameter *c* = 0.003 (estimate of the mean number of centiMorgans per kb in euchromatic regions of the genome) and number of clusters equal to 20 (maximum number of groups in which the local haplotypes will be grouped). The analysis was based on the hidden Markov model (Scheet and Stephens, 2006), using the LD between the SNPs within the haplotype blocks. The accuracy of the imputation was estimated by the concordance rate (proportion of correctly imputed genotypes), which 10% of the genotypes were randomly masked followed by imputation and comparison with the true results.

### Genomic Distribution of Markers

To evaluate the marker coverage on the genome, all sequences were aligned to the *P. vulgaris* reference genome (Schmutz et al., 2014) using the BLASTN *E*-value ≤ 1E-10 (Altschul et al., 1997) and the best hit genomic location. The distribution of the markers in the genome was represented using R v. 3.0.1 (R Core Team, 2015). SnpEff version 4.2 (Cingolani et al., 2012) was used to annotate variants in the genome assembly based on their targeted regions and predicted coding effects, as described by Valdisser et al. (2017).

### Linkage Disequilibrium and Haplotypes Blocks

The markers were tested for pairwise LD by using the quadratic coefficient of correlation (*r*^2^), corrected for the bias due to population structure (*r*^2^_*s*_), relatedness (*r*^2^_*v*_), and for both (*r*^2^_*SV*_) using the LDcorSV package (Mangin et al., 2012; Desrousseaux et al., 2017). The genetic relationship matrix was calculated using the R-package rrBLUP (Endelman, 2011) using the method proposed by VanRaden (2008). The estimation of the LD extension throughout the genome was carried out using the non-linear model proposed by Hill and Weir (1988), adjusted with *nls* function of the R software v. 3.0.1 (R Core Team, 2015). Haplotype blocks were identified using the Haploview 4.2 (Barrett et al., 2005) from the total set of markers (11,870) based on the confidence interval described by Gabriel et al. (2002). Heterozygous loci were eliminated from the analysis.

### Genetic Diversity and Structure

We used the data of the LD-pruned markers (LE; keep only markers with *r*^2^ < 0.8 inside a window of 50 kb) using the Plink v. 1.07 (Purcell et al., 2007), with minor allele frequency (MAF) > 0.05 and call rate ≥ 95% to obtain the estimates of genetic diversity and population structure. The GenAlEx6 software (Peakall and Smouse, 2012) was used to obtain the estimates of observed and expected heterozygosity (genetic diversity of Nei), probability of identity (PI), and exclusion (PE) by means of the multilocus option. The population structure was heuristically inferred considering subpopulation numbers (*K*) ranging from 1 to 10, using 20 interactions for each *K*. The models for each *K* assumption were fitted in Bayesian approach using 200,000 Markov Chain Monte Carlo with discard of 100,000 interactions due burnin Period (Pritchard et al., 2000).

The method reported by Evanno et al. (2005) using Structure Harvester v. 0.6.93 (Earl and vonHoldt, 2012) was used to estimate the most likely K explaining the population structure, followed by the identification of the best alignment to the replicate results of the cluster analysis using the CLUMPP software (Jakobsson and Rosenberg, 2007). The organization chart was generated in R v. 3.1.3 (R Core Team, 2015). A phylogenetic tree reconstruction was carried out with the RAxML v. 8.2 (Stamatakis, 2014) using both rapid bootstrap algorithm and search for best scoring in combination with an ML search. A GTR model of nucleotide substitution with a gamma model of rate heterogeneity was applied, and branch support was determined by 10,000 bootstrap replicates. Tree ETE Toolkit 3.0 (Huerta-Cepas et al., 2016) was used to visualize the phylogenetic tree with RAxML v. 8.2.

### Markers Under Signature of Selection (Outliers)

The outlier markers were detected using the hierarchical method of Excoffier et al. (2009) implemented in Arlequin v. 3.5.2.2 (Excoffier and Lischer, 2010), which identified outlier loci by comparing the levels of genetic diversity and differentiation between populations. The hierarchical island model was simulated assuming 100 demes with 20,000 simulations to generate an *F*_*ST*_ joint distribution versus heterozygosity. Those loci that fall outside the 95% confidence interval were considered outliers and were annotated using the Phytozome database (Schmutz et al., 2014).

### GWAS Analysis

The GWAS based on individual analysis of markers was performed in the Tassel software version 5.2.44 (Bradbury et al., 2007) using the MLM module, considering the markers and the population structure groups as fixed-effect factors and the genetic background as a random-effect factor with correlated effect as represented in the kinship matrix. The kinship matrix was estimated using the identity-by-state algorithm in the Tassel software. The population structure matrix (Q matrix) was obtained using the Structure software. The significance of associations between markers and phenotypic traits was evaluated using the false discovery rate (FDR) method, as implemented in the Qvalue package version 1.0 (Storey, 2002) of R v. 3.0.1 (R Core Team, 2015).

The GWASs using gene and segment-based models were performed to leverage the aggregated effect of sets of SNPs, given that it is higher than the individual effect of the markers. Two approaches were used: (1) a gene-based model using a gene region defined as 50 kb of UTRs of a gene, and (2) a segment-based GWAS model using segments of 100 kb (close to the LD extension value). These approaches were conducted using fastBAT (Bakshi et al., 2016) in GCTA software (Yang et al., 2011). The proportion of the phenotypic variation explained by each window (*r*^2^) for the group of markers was estimated as described by Müller et al. (2019). The Bonferroni procedure was implemented to control for type I error at α = 0.05, and the Benjamini and Hochberg (1995) procedure was used to control for FDR at 5%. The multiple test corrections considered the total number of SNPs for the single SNP-based, total number of regions for the segment-based, and total number of genes for the gene-based GWASs.

## Results

### Productivity Components

Phenotypic data were successfully collected in five environments. In the drought stress periods, between May and August, there were no large variations between the years for the maximum and minimum temperatures (35.3 and 21.3°C, respectively), relative humidity (43.9%), and rainfall (0.13 mm). In general, crops during stress periods were grown under low relative air humidity and reduced rainfall (Supplementary Table 2).

The estimates of heritability (*h*^2^) for SW ranged from 0.85 (WS-2014) to 0.98 (NS-2016), and the GY ranged from 0.37 (WS; 2014) to 0.59 (NS; 2015) (Table 1). Selection accuracy was similar across all environments for both traits, with high values for SW (ranging from 0.93 in the WS-2014 and WS-2016 environments to 0.99 in the NS-2016 environment) and moderate values for GY (ranging from 0.71 in the WS-2016 to 0.76 in the NS-2015 environments). Overall, slightly lower estimates for the heritability coefficients and accuracy and higher coefficient of variation were observed for the experiments under water deficit stress.

In the control experiment (Table 1), the mean GY and SW were higher in 2014 (2,391 kg ha^–1^; 26.81 g) than in 2015 (1,187 kg ha^–1^; 22.24 g) and 2016 (1,947 kg ha^–1^; 26.77 g). Under drought, the highest estimates of GY and SW were obtained in 2016 (798.8 kg ha^–1^; 21.31 g), followed by 2014 (517.4 kg ha^–1^; 20.6 g). In the joint analysis, the adjusted average GY was 705.2 and 1,870 kg ha^–1^ in the environments with and without drought stress, respectively, indicating a reduction of 62% in production. For SW, the values were 23.23 and 27.78 with and without stress, respectively, showing a decrease of 16%.

**FIGURE 1:**
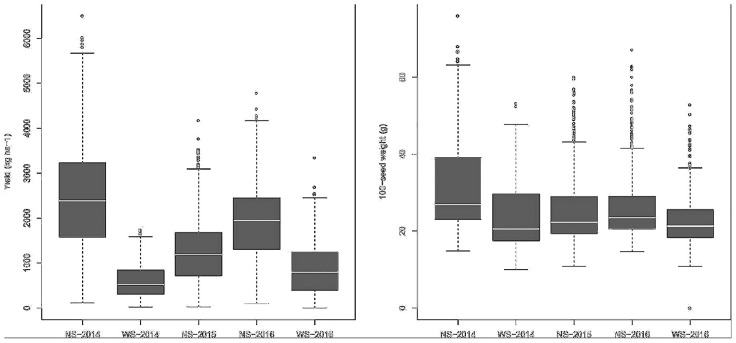
Box plots from the BLUP-adjusted values for yield and 100-seed weight taken during the 3 years of experimentation, with (WS) and without (NS) drought stress.

Considering the cultivated and landrace germplasms analyzed separately, the mean GYs were 1,885.74 and 1,892.37 kg ha^–1^ in the irrigated environment, and 708.21 and 713.53 kg ha^–1^ under drought, respectively. For SW, the averages were 24.17 and 22.57 g under irrigation and 21.11 and 20.45 g under drought for cultivated and landrace germplasms, respectively. The GY and SW box plot charts showed a great dispersion of data in both the control and drought experiments (Figure 1).

Based on the GY values, the genotypes were classified into 10 most and least productive accessions, with and without stress (Table 2 and Supplementary Figure 1). Under irrigation, the most productive group consisted of three cultivars/lines and seven landrace varieties, which on average produced 2,260 kg ha^–1^, whereas the least productive was composed of six landrace varieties and four cultivars/lines, which produced on average 1,552 kg ha^–1^, that is, a reduction of 31%. Under the water deficit, the most productive group was represented by four cultivars/lines and six landrace varieties (mean of 893 kg ha^–1^) and the least productive by six landrace varieties and four cultivars/lines (mean of 563 kg ha^–1^), with a reduction of 37%. Three genotypes (Ouro Negro, CF200012 and CF800113) were consistently classified as the 10 most productive in both conditions of NS (2,314, 2,239, and 2,262 kg ha^–1^) and WS (973, 873, and 871 kg ha^–1^).

**TABLE 2:** Ranking of the 10 genotypes that presented higher and lower yields (GY) obtained through BLUPj in the experiments with (WS) and without water restrictions (NS).

**Groups**	**WS**		**NS**	
	**Genotype ID**	**GY (kg ha^–1^)**	**Genotype ID**	**GY (kg ha^–1^)**
**10+**	Ouro Negro* (lines/cultivar)	973	CNF008845 (lines/cultivar)	2,371
	BRS Pontal (lines/cultivars)	905	Ouro Negro* (lines/cultivar)	2,314
	CF871226 (Landrace)	899	CF240056 (Landrace)	2,286
	CNF007143 (lines/cultivar)	894	CNF011036 (lines/cultivar)	2,275
	CF250002 (Landrace)	889	CF800113* (Landrace)	2,262
	CNF007050 (lines/cultivar)	886	CF200012* (Landrace)	2,239
	CF890223 (Landrace)	881	CF840600 (Landrace)	2,225
	CF200012* (Landrace)	873	CF200048 (Landrace)	2,216
	CF800113* (Landrace)	871	CF870030 (Landrace)	2,208
	CF860105 (Landrace)	863	CF800110 (Landrace)	2,201
**10 -**	CF840275^§^ (Landrace)	537	CNF001611 (lines/cultivar)	1,501
	CNF007770^§^ (lines/cultivar)	556	CF200075 (Landrace)	1,528
	CF840650 (Landrace)	557	CF840747 (Landrace)	1,545
	CNF007646 (lines/cultivar)	559	CNF006978 (lines/cultivar)	1,555
	CNF005887 (lines/cultivar)	562	CNF007770^§^ (lines/cultivar)	1,561
	CF890110 (Landrace)	564	CF840275^§^ (Landrace)	1,561
	CF240032 (Landrace)	568	CF840543 (Landrace)	1,561
	CF240016 (Landrace)	574	CF810080 (Landrace)	1,564
	CNF005482 (lines/cultivar)	575	CNF004121 (lines/cultivar)	1,570
	CF800049 (Landrace)	575	CF841226 (Landrace)	1,577

*Genotypes with higher yield in environments with and without water deficiency. ^§^Genotypes with lower yield in environments with and without water deficiency.

### DArTseq and CaptureSeq Genotyping

Genotyping of 339 Mesoamerican bean accessions using DArTseq technology provided 24,484 markers, of which 11,862 were SNPs and 12,622 were polymorphic SilicoDArT markers. For the SNPs, the mean proportion of homozygotes and heterozygotes was 0.99 and 0.01, respectively, whereas the mean of minor allele frequency estimate was 10.16%. Regarding data quality, reproducibility was high, with values ranging from 94.5 to 100% (average of 99.85%), and the mean proportion of missing data per marker was 6% (mean call rate of 94%). Using CaptureSeq technology, 11,989 SNPs were generated from 3,478 probes evaluated in 175 genotypes, with an average of 3.45 SNPs per probe.

In total, 36,473 markers were generated, of which 17,375 SNPs (11,638 and 5,737 derived from DArT and CaptureSeq, respectively) were successfully imputed for 339 genotypes (accuracy of 97%), resulting in 11,870 useful markers (MAF ≥ 5% and missing data < 5%) (Table 3).

**TABLE 3:** Distribution of SNPs and SilicoDArT on individual chromosomes of *P. vulgaris*.

**Chrm**	**Imputed markers^ε^**				**Total of markers**				Chr size (kb)*	**Average no. of total markers/Mb**
	**SNP_ DArT**	**SNP_ CaptSeq**	**DArT**	**Total**	**SNP_ DArT**	**SNP_ CaptSeq**	**DArT**	**Total**		
1	515	291	255	1,061	1,066	914	1,072	3,052	52,183.5	58.49
2	610	286	312	1,208	1,388	956	1,426	3,770	49,033.7	76.89
3	539	265	318	1,122	1,190	909	1,340	3,439	52,218.6	65.86
4	488	345	395	1,228	828	1,471	1,015	3,314	45,793.2	72.37
5	353	186	197	736	790	785	847	2,422	40,237.5	60.19
6	472	198	215	885	980	808	948	2,736	31,973.2	85.57
7	459	273	267	999	1,057	953	1,097	3,107	51,698.4	60.10
8	831	574	484	1,889	1,317	2,005	1,468	4,790	59,634.6	80.32
9	363	179	180	722	1,065	786	1,016	2,867	37,399.6	76.66
10	369	244	242	855	829	827	912	2,568	43,213.2	59.43
11	492	325	348	1,165	1,121	1,470	1,288	3,879	50,203.6	77.27
Scaffolds	—	—	—	—	231	105	193	529	—	—
**Total**	**5,491**	**3,166**	**3,213**	**11,870**	**11,862**	**11,989**	**12,622**	**36,473**	**513,589.1**	**71.02**

^ε^MAF ≥ 0.05; call rate > 95%. *Schmutz et al. (2014). Chrm., chromosome; CaptSeq, CaptureSeq.

### Genomic Distribution

All markers evaluated (36,473) were aligned to the common bean genome (Schmutz et al., 2014), of which 529 were in scaffolds (Table 3 and Supplementary Figure 2). Among the SNPs, transitions (Ts) were the most abundant (55%), with the transition from cytosine to thymine being the most frequent polymorphism (3338 DArT SNP and 1,706 CaptureSeq SNP), whereas transversions (Tv) were identified in 45% of the SNPs. The Ts/Tv (transition/transversion) rates observed were 1.22 and 1.27 in DArT SNP and CaptureSeq SNP, respectively. Overall, the mean number of markers per chromosome was approximately 3,268, with an average of one marker every 14,081 bp (71.02 markers/Mb) estimated.

### Linkage Disequilibrium Analysis

**FIGURE 2:**
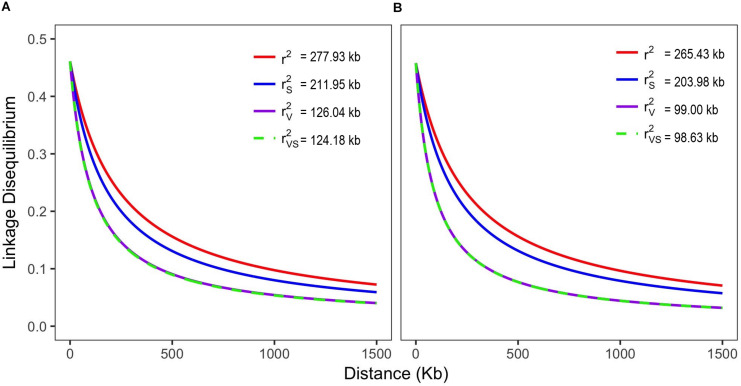
Genome-wide pattern of linkage disequilibrium (LD) decay in the lines/cultivars **(A)** and landrace **(B)** Mesoamerican germplasms represented by decay curves without any correction (*r*^2^), adjusted for population structure (*r*^2^_*s*_), relatedness (*r*^2^_*v*_) and for both (*r*^2^_*sv*_).

The mean distance between markers along the chromosomes was 21.43 Mb, with minimum and maximum distances of 1 bp and 63 Mb, respectively. For the LD analysis, more than 692,000 *r*^2^ pairwise estimates were calculated per chromosome. In general, the *r*^2^ distribution showed a rapid LD decay when analyzing all genotypes (339) simultaneously, as the physical distance increased (*r*^2^_*SV*_ ∼0.23). On the basis of the *r*^2^ model (with no correction for the population structure), LD extended over 237 kb, ranging from 158 kb on chromosome 4–436 kb on chromosome 9. When the corrections for structure and relatedness were applied (*r*^2^_*sv*_), the LD extension dropped by ∼70%, reaching the value of 63.38 kb (Figure 2 and Supplementary Table 3). Reduced LD extension was observed on chromosome 8 (32.48 kb) and greater on chromosome 9 (172.78 kb). Considering only the landraces, the LD extension was estimated to be at 98.63 kb, whereas for the breeding lines, it was 124.18 kb.

### Haplotype Blocks Distribution

A total of 1,548 haplotype blocks were identified, ranging from 83 (chromosome 9) to 242 (chromosome 8). A total of 9,873 markers (83.18%) were located in these blocks, with a mean of approximately six markers per block. Chromosome 3 (86.72%) and chromosome 9 (60.66%) had the highest and lowest percentage of markers/block, respectively. The mean block size was 21,266.64 kb, with the largest block found in chromosome 8, comprising 33,804.67 kb and 1,605 markers, and the smallest block was identified on chromosome 7, with 13,949.93 kb and 835 markers. The maximum and minimum haplotype frequency was 0.95 and 0.01, respectively, with the most frequent being located on chromosomes 7 and 8. The blocks covered, on average, 45.55% of the total genome (Supplementary Table 4). For the landrace accessions, 1,329 blocks were identified within the 11 chromosomes, with 8,977 markers located within these blocks (87.73%), with a mean of 6.75 markers per block and sum of blocks size 228,534 kb (mean of 171.96 kb per block). For the cultivated accessions, 1,358 blocks were identified in the 11 chromosomes with a total size of 222,286 kb (mean of 163.69 kb per block), of which 84.4% (8,534) of the markers were located within blocks with a mean of 6.28 markers per block. The highest number of blocks was observed on chromosome 8 for both strata of germplasms (213 for landraces and 220 for lines/cultivars).

### Genetic Diversity and Structure Analysis

These analyses were conducted using the set of 4,941 markers in linkage equilibrium obtained from LD pruning. The average estimates of *H*_*E*_ for the Mesoamerican Brazilian germplasm (*n* = 339) was 0.277 (±0.002), whereas for the landraces (*n* = 224) and lines/cultivars (*n* = 115), they were estimated at 0.262 (±0.002) and 0.285 (±0.002), respectively. With regard to *H*_*O*_, *F*_*IS*_, and *F*_*IT*_ indices, the values were 0.023 ± 0.001, 0.917 ± 0.001 and 0.921 ± 0.0031, respectively, for the entire set of samples (Supplementary Table 5). A high number of polymorphic SNPs was identified for the landraces (99.98%) and lines/cultivars (99.84%), and a moderate genetic differentiation (*F*_*ST*_), according to Hartl and Clark (1997), was observed between the germplasm groups (0.051 ± 0.001). Considering the marker informativity, SNP markers were slightly superior to SilicoDArT at estimating the parameters of mean gene diversity (*H*_*E*_) and genetic differentiation (F*_*ST*_*). Through analysis of molecular variance (AMOVA), greater among accessions variation (74%–95%) than among germplasm groups (5%) was observed.

The probability that two unrelated individuals share the same genotype (PI) was null (zero), reaching the minimum value of 1,5342 × 10^–140^ for SNP from CaptureSeq and landrace germplasm. To achieve a PI < 0.0001, a maximum of 34 SNPs were required to differentiate genotypes within cultivars and 38 within landraces. The PE estimated was high, practically reaching 100% of probability of exclusion using panels ranging from 60 to 77 markers based on SilicoDArT and SNP DArT, respectively.

**FIGURE 3:**
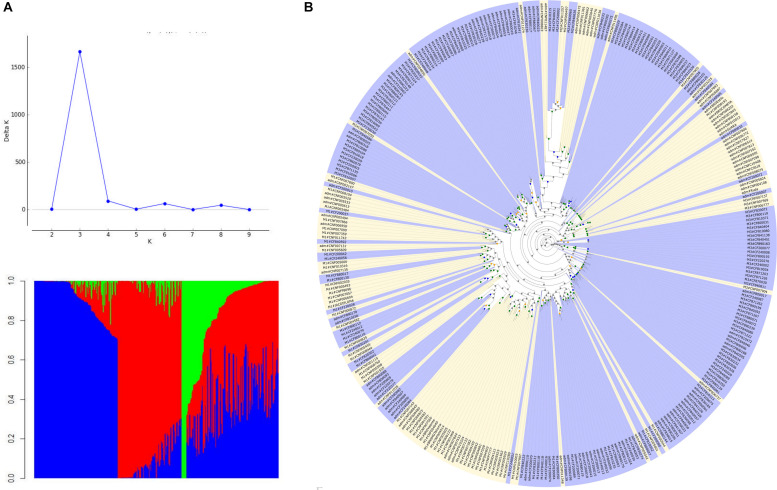
Population structure and best scoring maximum likelihood tree based on the genotyped data sets. **(A)** Population structure for the Mesoamerican germplasm (*K* = 3) showing, predominantly, lines/cultivars (M1, red), landraces with large grains (M2, green), landraces with a wide variation of commercial grain types (M3, blue); **(B)** phylogenetic tree where the range of branch support was depicted at the nodes (gray dots: 0–60; orange dots: 60–70; blue dots: 70–85; and green dots: 85–101); landrace germplasms are marked in blue and the lines/cultivars in yellow; the names of genotypes are shown on the top of the corresponding clades and integrate information on to which structure group they belong.

Structure analysis suggested *K* = 3, subdividing the genotypes into three groups: M1, M2, and M3. The M1 group consisted of a cluster of 88 accessions, the majority of black commercial grain type (70%), being 61% cultivars/lines and 39% landraces. In the M2 group, with only seven accessions, there was a predominance of landrace germplasm (71%), and the majority (71%) had a large grain size. The M3 group consisted of 116 accessions, of which 95% were landrace varieties and with a wide variation of commercial grain types, with predominance of Mulatinho and Carioca grains (39%). The M1 group was revealed to be more diverse (*H*_*E*_ = 0.229), followed by the M3 (*H*_*E*_ = 0.198) and M2 groups (*H*_*E*_ = 0.045). A total of 128 accessions (38%) were considered as resulting from multiple ancestry (admixture) and presented the highest value of diversity (*H*_*E*_ = 0.298). The results of ML best tree and bootstrap searches on the genotyped data sets are summarized in Figure 3. The phylogenetic analysis showed a strong tendency to group the accessions according to the germplasm type, landraces, and lines/cultivars. These results were in accordance with the Structure analysis.

### SNPEff and Outlier Markers

A total of 9,838 effects were predicted for 3,582 SNPs, considering the information on the location of all isoforms, genic, and intergenic regions in the common bean genome (Schmutz et al., 2014). The predicted effects were of modifier type (80.87%), low impact (13.03%), moderate impact (5.71%), and high impact (0.39%). Most SNPs with predicted effects were observed in genic regions (5,699), of which 30.18% and 28.91% were observed within exons and introns, respectively, with the remaining in non-translated regions. In genic flanking sequences (5-kb window), 4,139 effects were identified, of which 55.81 and 44.19% occurred in downstream and upstream regions, respectively. From the 22 SNPs predicted with high impact (Supplementary Table 6), 18 were annotated in different functions, such as kinases, transcriptions factors, and cellulose synthase, among others.

A total of eight loci deviated from the neutrality hypothesis (outliers) when comparing the landraces and cultivars/lines groups (*p* < 0.05), with five and three being present on chromosomes 7 and 8, respectively (Supplementary Table 6). Of these, six are SNPs (four DArT SNPs and two CaptureSeq SNPs), and two are SilicoDArT. Based on SNPEff analysis, 17 putative effects were predicted for six of the eight outliers, of which 5.88% were low-impact, 17.65% moderate, and 76.47% modifier type. The six outliers SNPs have effects on gene regions (11.76% on introns and 23.53% on exons), on intergenic regions (5.88%), and on regions flanking genes (5-kb window), 47.06% on downstream region and 11.76% on the upstream region. The Tv/Ts rate of the outliers was of 2. From the eight outliers’ loci, five were on genes described as salt stress response and have antifungal activity, tyrosine kinases, CCCH zinc finger protein, lipid transfer protein, and DNA helicase.

### GWAS Using Single SNP Model

**FIGURE 4:**
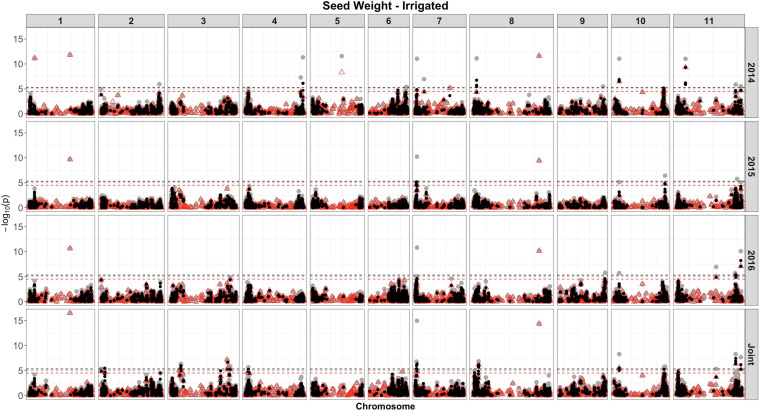
Manhattan plot of the associations for SW for common bean, under irrigation, using a single-based GWAS (gray circle), segment-based GWAS (red triangle), and gene-based GWAS (black circle) based on the role set of experimental data. Gray, red, and black lines indicate Bonferroni-corrected threshold with an experimental type I error rate at α = 0.05.

**FIGURE 5:**
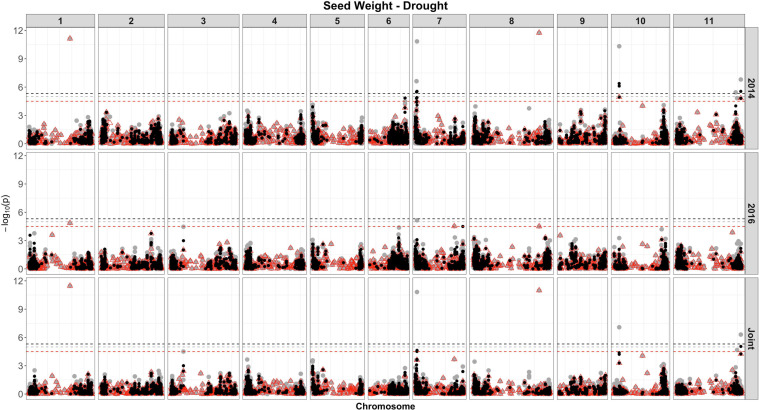
Manhattan plot of the associations for SW for common bean, under drought, using a single-based GWAS (gray circle), segment-based GWAS (red triangle), and gene-based GWAS (black circle) based on the role set of experimental data. Gray, red, and black lines indicate Bonferroni-corrected threshold with an experimental type I error rate at α = 0.05.

**FIGURE 6:**
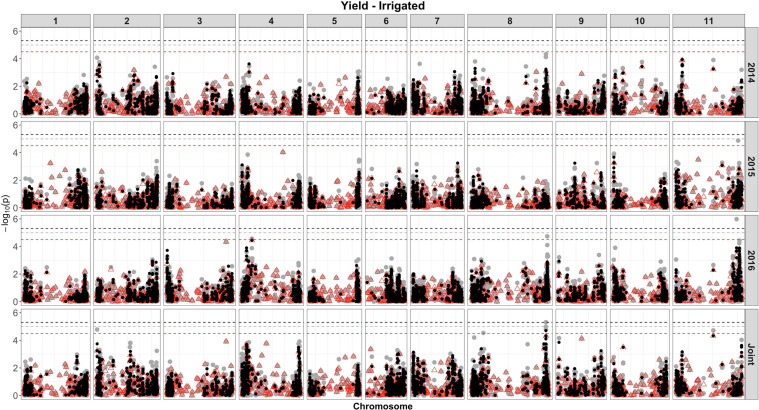
Manhattan plot of the associations for GY, under irrigation, using a single-based GWAS (gray circle), segment-based GWAS (red triangle), and gene-based GWAS (black circle) based on the role set of experimental data. Gray, red, and black lines indicate Bonferroni-corrected threshold with an experimental type I error rate at α = 0.05.

**FIGURE 7:**
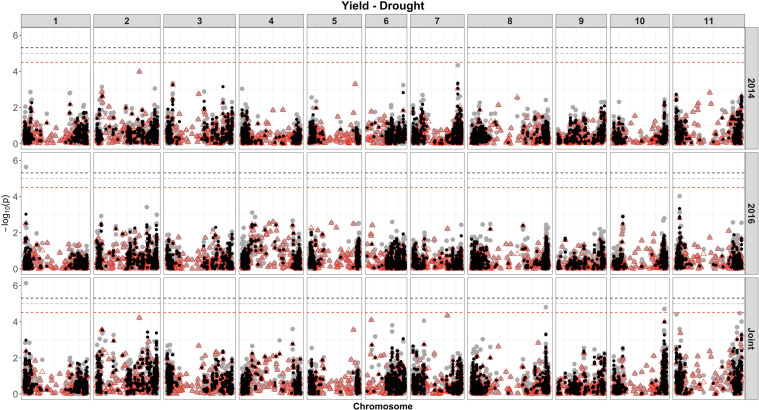
Manhattan plot of the associations for GY, under drought, using a single-based GWAS (gray circle), segment-based GWAS (red triangle), and gene-based GWAS (black circle) based on the role set of experimental data. Gray, red, and black lines indicate Bonferroni-corrected threshold with an experimental type I error rate at α = 0.05.

Based on single-SNP model GWASs, a total of 210 marker-trait associations were detected for the SW (Figures 4, 5, gray circles) and GY (Figures 6, 7, gray circles) in the field experiment conducted under irrigation and drought for the 3 consecutive years.

For SW, irrigated and drought experiments, a total of 177 associations were identified, of which 61 were detected over more than two analyses and presented similar effects across experiments (Figures 4, 5). The number of associations for SW in irrigated conditions was 173 (Supplementary Table 7), ranging from 24 on chromosome 3, to 4 on chromosome 5, and showing the presence of nine clusters of QTLs. Based on the joint analysis, QTLs under irrigated conditions were identified (123), with phenotypic variance accounted for by these markers ranging from 2.82 to 22.5%. Of the 173 QTL identified, 115 were exclusive, and 58 were common across experiments (Figure 4).

For SW, under drought, of the 18 QTLs identified (Supplementary Table 7 and Figure 5), 14 were common across irrigation treatments, one across the same year of experimentation (SNP 8212321), two across 2 years of experimentation (DArT 3382165 and SNP 3377127), and one QTL found in four experiments, across treatments in all years of experimentation (SNP 1139472, *r*^2^ = 9.56%). This last marker that falls apart 1.5 kb of a gene encoded a protein involved in transport process (mechanosensitive ion channel). The phenotypic variance ranged from 5.35 to 20.35%. Overall, three associations were consistently identified in all 3 years of experimentation and joint analysis (SNPs 3378295, 3380258, and 3384387).

For GY, a total of 33 QTLs were identified, in the two irrigation treatments, across 10 chromosomes, and the phenotypic variance ranged from 4.42% to 12.95% (Supplementary Table 8 and Figures 6, 7). Most QTLs (23 of 33) presented a negative alternative allele’s effect over the target trait. Among all, four QTLs were consistently identified under more than one growth condition and presented similar effects over the trait. These four included the SNP 1572065, detected by both drought in 2016 and joint analysis that accounted for 11.56 and 7.93% of the phenotypic variance, respectively, and DArT 3371424 identified by joint analyses in both drought and irrigated experiments, which explained 6.05 and 6.20% of the phenotypic variance, respectively. These markers were located within putative gene sequences involved in pre-mRNA maturation (polyadenylate-binding protein) and nitrogen assimilation (asparagine synthase), respectively. From the seven QTLs identified on chromosome 8, four were detected in the marker interval 60713295–61898258 (∼1.2 Mb) and formed a QTL cluster, placed on different haplotype blocks, with the phenotypic variance accounted by these markers ranging from 5.85 to 9.55%.

### Segment-Based Models

For the segment-based GWASs, 1,588 segments of size 100 kb were tested. Of the 105 associations identified for the SW, under irrigation (Figure 4, red triangle; Supplementary Table 9), a higher number of associations were detected in the experiment conducted in 2016 (40), followed by 2014 (36) and 2015 (11). For the analysis combining the whole data set, a total of 58 associations were identified, with 12 being common between, at least, two experiments. The proportion of the phenotypic variation ranged from 0.0074% (SNP 16648859) to 32% (DArT 3376862). Overall, four associations were consistently identified in all 3 years of experimentation and joint analysis (SNPs 3384387, 3383169, 3380258, and 3378295), explaining on average 8–21% of the phenotypic variance. These markers were found within gene sequences with diverse putative functions, such as binding pectin polymers in the cell wall, DNA/RNA binding and protein interactions, nitrogenase activity, and regulation of photosystem II, respectively. In addition, these same four markers were shared between SW evaluated under drought and irrigated conditions. All associations were comparable to those obtained using single-model GWASs, and 12 QTLs were not shared between GWAS methods.

For the SW evaluated under drought (Supplementary Table 9 and Figure 5), eight associations were identified. Of these, two (SNP 3378295 and SNP 3380258) were consistently identified over the three independent analyses (2014, 2015, and joint analysis), and two (SNP 3383169 and DArT 3382165) were detected in the experiment of 2014 and joint analysis. The proportion of the phenotypic variation ranged from 0.038% (SNP 3377757) to 32% (SNP 3378581), and the two markers consistently identified over experiments explaining, on average, 20% of the phenotypic variance. The most significant regions declared were placed on chromosomes 1 (SNPs 3380258) and 8 (SNP 3378295). Two markers on chromosome 6 placed at the same interval (28842624 to 28934587 bp), corresponding to a transcribed region of a NADH dehydrogenase (Ubiquinone), explained the highest proportion of the genetic variation (32%) under drought (SNP 3378581) and irrigation conditions (DArT 3376862).

For the GY, overall, nine significant regions at the genome-wide level were captured (five under irrigation and four under drought), of which all QTLs were detected by single-model GWASs (Figures 6, 7, red triangle and Supplementary Table 10). Combining the data of all experiments increased the power to detect significant associations, and using this approach, eight (four under drought and four under irrigated conditions), of the nine associations, were identified. The most significant region declared for GY, under irrigation, contains two SNPs (S04_8176704 and 3369849), placed on chromosome 4 and within a ubiquitin gene. The association that explained the highest proportion of the genetic variation under drought (SNP 3383887, 17%) was located physically close (∼4 kb) to transcripts related to DNA transcription and RNA synthesis.

### Gene-Based GWASs

Of the 27,012 genes restricted to chromosomes (including genes only in chromosomes 1–11) in the *P. vulgaris* genome v. 2.1 (Schmutz et al., 2014), 10,163 were considered as gene sets in our analysis as they contained SNPs targeted by the DArTseq and CaptureSeq approaches. For the GY trait, no significant association (FDR at 5%) was detected using this approach, even combining the data set by gene-based joint GWASs.

For the SW trait, under irrigation (Figure 4, black circle and Supplementary Table 11), 651 significant genes were identified for the three experiments and joint analysis, placed on 220 distinct start- and end-SNPs intervals, spanning all chromosomes, except on the chromosome 5, with a large number of associated genes (101) on chromosome 11. The number of SNPs by gene ranged from 2 to 20 (chromosome 11, interval containing the gene Phvul.011G194900), and putative functions were attributed to 532 genes. As expected, most gene associations (∼70%) were also detected at the same significant genomic regions identified by segment-based and single-model SNP GWASs. These genes explained proportions of the phenotypic variations that ranged from 0.24 to 27%, and blocks of genes that explained more than 20% of the phenotypic variance were placed in all, except on chromosomes 1, 5, and 8.

A total of 35 genes placed on 4 chromosomes (6, 7, 10, and 11) were significantly associated with SW at drought, restricted to the experiment conducted in 2014 and the joint analysis, of which 12 were common between these analyses (Figure 5, black circles and Supplementary Table 11). The number of SNPs contiguous with the genes ranged from two to four. Seven genes were placed on chromosome 6, within an interval of 65 kb (28897165 to 28962611), positioned at adjacent position (apart 4 Mb) and accounted for 26% of the phenotypic variance. On chromosome 7, 18 genes were identified (eight related with a putative kinase and oxidoreductase functions), spanning an interval of 444 kb; placed on four distinct intervals of SNPs; and explained proportions of the phenotypic variations ranging from 2.3 to 26%. As expected, some regions containing genes were common to those detected in the other analyses (single SNP- and segment-based models); however, many new associations have been revealed.

## Discussion

In common bean, there is a need to improve drought tolerance by selecting genotypes with high yield potential under stress and by introducing favorable alleles conferring these adaptations into cultivated germplasm. In this study, a high number of genetic markers and hundreds of Mesoamerican accessions with reduced genetic relationship and useful genetic variation were used for association analysis. The SW and GY data were recorded in field experiments conducted at the experimental station of Emater-GO, in Porangatu (Brazil), which historically presents few rain occurrences during the winter season requiring irrigation (Heinemann et al., 2007). Although this condition is suitable for drought phenotyping, dry season in Porangatu shows a decrease in accumulated radiation and a higher frequency of lower-than-optimal minimum temperature during the crop cycle (Heinemann et al., 2016), limiting lines/cultivar to express their full yield potential. Under irrigation, of the 10 genotypes with the best performance, seven were landraces, with yields similar to those of the cultivars and mostly coming from the South of Brazil (six out of seven). Embrapa started collecting landraces from several Brazilian regions in the 1970s, and the South region has been considered a valuable source of germplasm adapted for abiotic stresses (Vidigal-Filho et al., 2007; Pereira et al., 2009). In this study, water deficit resulted in significant reduction in productivity (by 62%), close to the yield reduction (by ∼70%) obtained by Smith et al. (2019) in the field evaluation of drought stress conducted at CIAT. Because increased yield under drought is one of the greatest challenges for the genetic breeding of common bean, one significant contribution of this study was to indicate genotypes with superior performance that could be explored through breeding. Among the 10 genotypes with outstanding yield performance under drought, six are landraces from Brazilian regions with contrasting edaphoclimatic conditions (Supplementary Figure 1). Moreover, three genotypes performed better with and without WS; two of them are landraces (CF200012 and CF800113). Somehow, the genetic diversity within these traditional germplasms gives the necessary plasticity to support an adaptive response to environmental variation, providing an advantage over improved varieties (Mercer and Perales, 2010; Dwivedi et al., 2016). This reinforces the fact that landraces remain a vital resource for contemporary plant breeding and demand permanent conservation and knowledge of their genetic variability (Villa et al., 2005; Azeez et al., 2018). Regarding the Ouro Negro cultivar, from Honduras, which has black grains and a normal cultivation cycle (80–100 days; Amaro et al., 2015), in addition to standing out for disease resistance (Valentini et al., 2017), it also has high capacity to fix nitrogen (Da Silva et al., 2017), combining a number of attributes for plant breeding.

The large-scale SNP discovery obtained by the DArTseq and CaptureSeq technologies provided an extensive genome coverage allowing the exploration of both the broad structural (DArTseq) and gene variation (CaptureSeq), providing a very powerful genomic tool to differentiate germplasm accessions and to carry out high-resolution association mapping. The 17,375 SNPs identified (11,870 with MAF ≥ 0.05 and call rate > 95%) were all placed on the *P. vulgaris* reference genome, supporting the analysis of population structure, LD, and identification of genomic regions under selection that have an impact on crop improvement research. For the SNPs from DArTseq, the estimates of marker reproducibility (99.8%) and call rate (94%) were close and consistent with those previously reported (Cruz et al., 2013; Ren et al., 2015), thus indicating the quality and reliability of this set of SNPs. Using the CaptureSeq, we reported the identification and genotyping of 3,166 SNPs located inside or in close proximity to 3,304 (65.42%) annotated protein-coding genes associated with drought tolerance (Pereira et al., 2020) along the common bean genome. Despite a reduced number of SNPs targeted, this strategy represents a real possibility to explore the polymorphism in gene regions of interest, enabling the mapping of the set of isoforms for association analysis. Imputation accuracy was robust (97%), favored by the homozygous nature of the common bean genome and the availability of reference genomes (Nazzicari et al., 2016). Overall, a single marker per 14 kb has been achieved, a slightly higher density than that previously reported (SNP/86 kb) by Valdisser et al. (2017) based solely on DArTseq. For the first time, a diverse group of Mesoamerican germplasms (*n* = 339) was genotyped with a wide genomic representativeness, providing a more uniform and realistic distribution of allelic frequency over the whole population. This large set of markers targeting the genome of elite cultivars and landraces from Mesoamerican gene pool enabled the discovery of alleles and useful haplotypes at different frequencies, providing opportunity for additional novel genetic discoveries, in addition to those reported in this study.

### Genetic Diversity

The pattern of diversity of common bean in secondary domestication centers has been reported worldwide, providing relevant information on the evolutionary history of this species (for review, see Bitocchi et al., 2017). In Brazil, although germplasms of Andean and Mesoamerican origins are present, there is a predominance of Mesoamerican accessions in both modern varieties and landraces, which presents high levels of gene diversity when compared to the Andean accessions (Burle et al., 2010; Cardoso et al., 2014; Valdisser et al., 2017). Considering all markers, the genetic diversity for the Mesoamerican germplasm (*H*_*E*_ = 0.277; *n* = 339) was higher than that previously estimated by Valdisser et al. (2017) (*H*_*E*_ = 0.168; *n* = 111) and Rodriguez et al. (2016), who considered only domesticated bean accessions (*H*_*E*_ = 0.157; *n* = 100), what certainly can be attributed to the larger sample size used in this study. Slightly higher *H*_*E*_ values obtained for lines/cultivars (*H*_*E*_ = 0.285; *n* = 115), compared with landraces (*H*_*E*_ = 0.262; *n* = 224), are probably due to the global representativeness of the cultivated germplasms (breeding germplasm including introductions from abroad) supporting the highest estimates of diversity, whereas the landraces originated only from Brazil. Despite a moderate genetic differentiation between germplasms (*F*_*st*_ = 0.051), we identified a genetic structure (*K* = 3) within this panel, predominantly, in accordance with the germplasm strata. The M1 represented a mixing of germplasms (*n* = 88; *H*_*E*_ = 0.229), with a predominance of cultivars/lines (61%) most from Brazil, and some came from Guatemala, El Salvador, and Colombia. A very similar scenario clustering a diverse germplasm from Mesoamerica was observed by Bitocchi et al. (2012) and Kuzay et al. (2020), representing a useful source of tropical adaptive variation. The M2 (*n* = 7; *H*_*E*_ = 0.045) group was separated from the larger groups (clustering landraces and abroad cultivar/lines from Colombia and Zambia), and although there is no clear explanation for this, aspects related to the domestication and adaptation process could support this finding (Carovic-Stanko et al., 2017). A more comprehensive pattern was observed for M3 (*n* = 116; *H*_*E*_ = 0.198), mainly formed by landraces (95%) representative of all Brazilian regions, which is an evidence that these accessions still retain much of their identity, a resource that remains to be better exploited by breeders in strategically planned breeding programs (Cortés et al., 2013). The common bean has been broadly adapted to the Brazilian territory (Burle et al., 2010), having a significant genetic value that cannot be neglected (Azeez et al., 2018). The greater between-accessions variation (≥74%) was observed, thus evidencing that the germplasm groups analyzed are not genetically homogeneous. In addition, the results showed that 38% of the accessions presented high level of admixture (*n* = 128; *H*_*E*_ = 0.298), showing the movement of alleles between germplasm groups, which contributes to the expansion of variability but should be carefully taken to prevent the loss of genetic identity of the landraces. Our results suggest that the Brazilian Mesoamerican core collection composed by different germplasm strata, established using morphogeographical data and multivariate analysis (Oliveira et al., 2011), covers a wide range of genetic variability and should be explored worldwide.

### Linkage Disequilibrium Analysis

The discovery of a large number of markers in Mesoamerican germplasms of diverse origins, with a good distribution along the chromosomes, enabled estimating LD extension with high reliability, which is very important in the association studies context (Song et al., 2015). The LD extension estimates with corrections for structure and relatedness (*r*^2^_*sv*_) showed a faster LD decay (63.38 kb), contrary to what was observed without correction (*r*^2^ = 237 kb), showing that modeling taking into account both the genetic structure and kinship strongly affects the association between markers. Our findings showed that the bias induced by genetic relatedness was stronger than that of population genetic structure itself, what was also observed by Diniz et al. (2019). This has impact on determining the number of markers that cover the common bean genome and, consequently, on practical applications such as studies of genomic selection, where the correct determination of a consistent marker sets would provide more robust prediction capacity (Liu et al., 2015). The decay reported here is higher (*r*^2^_*sv*_ = 63.38 kb; *n* = 339) compared with previous report with common beans (*r*^2^_*sv*_ = 130 kb; *n* = 111; Valdisser et al., 2017) for the Mesoamerican germplasm. In addition, a less extensive LD is likely a reflection of the broader genetic basis of the genotyped Mesoamerican germplasm, as it comprised a more diverse set of accessions from the Brazilian common bean core collection. In addition, as previously reported and expected, the lines/cultivars accessions showed a higher level of LD extension (124 kb), compared with the landraces (98 kb), certainly as a result of a smaller effective population size of the breeding populations, a consequence of the successive use of elite germplasm in forward breeding, which may reduce genetic diversity over time. Initiative has been reported in the past to introduce genetic diversity from the wild common bean into the breeding program (Acosta-Gallegos et al., 2007), but these resources are still underexploited.

Even using a large number of genome-wide markers, the haplotypes blocks represented only 45.5% of the common bean genome (containing ∼83% of the total markers) and had average size of 21 Mb. This coverage was considerably higher when compared with the study of Valdisser et al. (2017) for the Mesoamerican gene pool (12.2% with 5,531 SNPs), which was due to the expanded sample size and higher number of markers used in this study. In general, the markers were well-distributed along the chromosomes, and regions with a reduced number of markers (Supplementary Figure 2) were coincident with the chromosomal structure of common bean centromeric and pericentromeric regions (Schmutz et al., 2014). Thus, in order to activate higher haplotype completeness, in addition to the need to continue sampling genotypes from different origins, it is necessary to even increase the density of the markers throughout the genome. This can be obtained combining several methods for sequence variation discovery, such as the use of different restriction enzymes, sequencing of large genome fragments, and even sequencing of the entire genome, in addition to expansion of the representativeness of genotypes (Fu et al., 2016; Edge et al., 2017; Torkamaneh et al., 2019). Despite the great interest in the practical implications of these LD block structures for gene-mapping studies in common bean, an autogamous species, haplotype coverage is still low and should be considerably expanded in future studies with beans.

### Predicted Coding Effects and Outlier Markers

Data from this study indicated a smaller proportion of SNPs (0.39%) with predicted effects of high impact. These genes were associated with kinases, transcriptions factors, and cellulose synthase, among others, and the consequences of these changes are unclear. Certainly, the impact of these SNPs on the gene’s functionality could be buffered by the plasticity and robustness of genome architecture, expression, and regulation (Koonin and Wolf, 2010). Regardless of that, these results could be a useful resource for future experimental identification and provide interesting information for better common bean breeding. The analysis showed eight outlier loci placed on chromosomes 7 and 8, suggestive of an adaptive divergence between landrace and lines/cultivars. Regions under domestication placed on chromosome 7 in the Mesoamerican group were previously reported by Schmutz et al. (2014). In our study, the functional annotation showed outlier loci involved in important mechanisms that could have favored the adaptive process in domestication, such as genes associated with tolerance to biotic and abiotic stress placed apart ∼49 kb (protein kinases); a gene (DNA helicase) directly involved in cell cycle progression (Tsuchiya et al., 1998), which has impact on cell growth and cell division (Jones et al., 2017); lipid transfer proteins involved with a large number of biological process, defense signaling, and biotic and abiotic stress (reviewed by Salminen et al., 2016; D’Agostino et al., 2019); and a CCCH zinc finger family gene, which has an important role in plant developmental processes and response to biotic and abiotic stress (Pi et al., 2018). Altogether, these results suggest that the fixation of favorable alleles on these selective loci certainly conferred adaptive advantage affecting the breeding germplasm over time. For breeding purposes, these target loci are a valuable resource and could be exploited by creating and introgressing new variability into the cultivated germplasm and also be the starting point for applying gene editing strategies (Aranzana et al., 2019).

### GWAS Analysis

Several GWASs in common bean are based on analysis combining collections of Andean and Mesoamerican germplasms, providing a broad perspective over the common bean diversity (Perseguini et al., 2016; Raggi et al., 2019; Ojwang et al., 2019; Tigist et al., 2019). However, each gene pool has been domesticated for specific agronomic, morphological, and physiological and under diverse edaphoclimatic conditions (Rendón-Anaya et al., 2017). As a consequence, a change in allele frequency associated with the genetic factors is expected within the gene pools, and it may not be segregating or have a negligible effect in a different gene pool controlling the agronomic traits of interest. In this study, a diverse collection of Mesoamerican common bean germplasms was used, and although less genetic variation is expected, the detected associations should be more relevant to breeding purposes, because a large set of cultivated/elite germplasms has been evaluated, each with its individualized recombination history.

Seed weight is one of the yield components of common bean and, in general, is positively correlated with seed yield (Kamaluddin and Shahid, 2011; Balcha and Tigabu, 2015) and regulated by a complex series of genes during the growth period encompassing a large set of functions and biological processes (Zhang et al., 2015). The demand for development of productive common bean cultivars with adequate seed is increasing in importance, either for national or international market. Mesoamerican grains are representative of the most consumed bean types in Brazil, and the breeding programs have prioritized the selection of medium-sized (25–30 g) black and carioca beans, to meet the preferences of packaging companies and consumers (Carbonell et al., 2010). A relatively large number of SW QTLs mappings have been identified using different types and sizes of mapping populations, molecular markers, and mapping strategies (Pérez-Vega et al., 2010; Mukeshimana et al., 2014; Briñez et al., 2017; Sandhu et al., 2018; Kamfwa et al., 2015). To our knowledge, the highest number of QTLs for SW has been reported in this study (189), in addition to the genes identified by gene-based GWAS (651), and several previous studies have located SW QTL on the same chromosomes as described here.

A total of 185 exclusive QTLs were identified by single and segment-based analysis of SW under irrigation, of which 93 (50%) were common. In general, the proportion of phenotypic variance (*r*^2^) explained by the segment-based approach was typically higher (42% of the markers explained more than 10% of the phenotypic variance) than that of the single-based approach (8% explained more than 10%). This is expected as a result of the segment-based analysis, which combines interval mapping with association analysis to capture variance across the whole population, powered by the combined effect of several closely linked loci at the target locus (Resende et al., 2017). By segment-based approach, four QTLs were consistently identified across the experiments and joint analysis on chromosomes 1 (SNP3380258, maximum *r*^2^ = 21%), 7 (SNP3384387, 15%), 8 (SNP3378295, 20%), and 11 (SNP3383169, 17%), whereas by gene-based approach we identified 18 genes across the multiyear experiments and joint analysis positioned on chromosomes 7 (located at the 14.57 to 15.53 Mb) and 11 (at 48.51 and 52.81 Mb). From these genes, nine were characterized in diverse biological functions that act in a broad spectrum of cellular and physiological processes, which can provide useful reference for genetic improvement of SW in common beans. Among these, we highlighted genes associated with plant development and abiotic stress tolerance (Wang and Bouwmeester, 2017); genes that act regulating cellular processes (Mudgil et al., 2004); proteins determinant for signal transduction mediated by plant hormones (Schapire et al., 2006); and seed storage proteins (Dunwell, 1998), among others.

A total of 38 QTLs for SW were identified in, at least, two successive years, placed on all chromosomes, except 1 and 5. Based on GWAS analysis of Mesoamerican common bean panel, Moghaddam et al. (2016) also identified genomic regions associated with SW in the Durango and Jalisco subpopulation exactly coincident with the region identified on chromosome 8 in this study (Phvul.008G013300) and very close to the regions identified for chromosomes 6 and 10. Transcript Phvul.008G013300 encodes a subtilisin-like serine protease that plays roles in plant development and signaling cascades. Previous reports have also identified QTL for seed size on chromosome 9 (markers ss715646851 and ss715646847, Hoyos-Villegas et al., 2017) located at ∼16.6 Mb (*r*^2^ = 4% for each SNP), under both irrigated and rain-fed conditions based on a Mesoamerican gene pool (*N* = 96), and quite similar to the QTLs identified in this study (chromosome 9, ∼17 Mb), providing evidence of a consistent region controlling SW across different environmental conditions and genetic backgrounds.

Among the yield components, SW has a large impact under drought, being affected by several physiological mechanisms, such as oxidative damages, photosynthate remobilization, nitrogen assimilation, and plant growth, among others (Sehgal et al., 2018; Teran et al., 2019). The impact of drought on common bean for SW in a field environment conducted at CIAT resulted in a reduction by ∼25% (Smith et al., 2019). In this study, drought stress reduced SW by 16%. Regarding the GWAS analysis, a reduced set of associations was identified for SW under drought, compared with the irrigated condition, and the higher number of associations was identified using gene-based approach (35). The top associated markers/genes were placed on chromosomes 6 and 7 comprising 14 genes, of which resistance gene was highlighted (Toll–interleukin resistance), located at 28.91 Mb, identified by both segment and gene-based analyses (*r*^2^ from 26 to 32%, respectively). Several genes in the vicinity of those association signals were identified by gene-based analysis, providing additional and complementary biological information that would not have been easily observed with SNP-based GWAS alone. Several SW QTLs under drought were previously identified on chromosome 1, at the 3.3- and 47.7-Mb position (Trapp et al., 2015) and at the interval of 54.88–76.87 Mb (Briñez et al., 2017, an *r*^2^ = 17.32%); on chromosome 3 at 23–33 Mb and chromosome 7 at 150 Mb (Mukeshimana et al., 2014); and on chromosome 9 at ∼16.6-Mb position (Hoyos-Villegas et al., 2017), among others. These QTLs vary both in relation to the mapped chromosome, as well as in to the position within the chromosomes compared to this study, indicating a great diversity of regions in the bean genome associated with SW in the presence of drought.

Interestingly, three QTLs on chromosomes 1 (SNP 3380258 at 33 Mb), 7 (SNP 3384387 at 1.5 Mb), and 8 (SNP 3378295 at 54 Mb) and 28 genes placed on chromosomes 7, 10, and 11 were identified in both irrigation conditions (WS and NS), suggestive that these QTLs have a constitutive effect on SW. These genes were classified functionally into several groups, including the following: (1) kinases, which play an important role in the regulation of cellular functions, including carbohydrate metabolism, growth, and differentiation, among others, having an impact on GY (Zhang et al., 2012; Shankar et al., 2015); (2) genes involved in the regulation of cell-wall polymers, having a structural role in plant growth and stress defenses (Gao et al., 2017); (3) genes that encode proteins that play a role in the vesicle budding from the endoplasmic reticulum (ER) and ER–Golgi protein trafficking, which could be involved in carbohydrate translocation to seed (Wang et al., 2016); (4) proteins involved in plant hormone signaling, such as gibberellin, cytokinin, and auxin responses, as well as ethylene biosynthesis, which mediate regulation of plant growth, development, and consequently GY (Schapire et al., 2006; Nadolska-Orczyk et al., 2017); and (5) genes that have roles in regulation of organelle transcriptome and biogenesis (Manna, 2015). In summary, we can see important genetic mechanisms under drought of great importance for SW, thus providing a considerable amount of information. From the perspective of practical and applied breeding, this large set of QTLs identified under irrigation and drought is challenging and difficult to be selected and manipulated because of their genetic complexity. Based on these results, there is strong evidence that the approach of genomic selection might be more appropriate, because it would take into account the effects of all markers and allow the selection based on genomic breeding values (Meuwissen et al., 2001).

For the GY trait, under irrigation, 25 QTLs were identified by the single- and segment-based approaches, placed on chromosomes 2, 3, 4, 8, 9, and 11. However, no QTL for GY was consistent across experiments, and only two QTLs (3371424 and 3370686) identified by single-model approach were consistent across water treatments. Several QTLs in this study were found on chromosomes previously identified as containing QTLs for yield conducted in several environments and genetic backgrounds. Based on genetic linkage analysis, QTLs for yield have been reported throughout chromosomes 1, 2, 3, 4, 5, 7, 8, 9, 10, and 11 (Tar’an et al., 2002; Beattie et al., 2003; Blair et al., 2006; Wright and Kelly, 2011; Checa and Blair, 2012; Mukeshimana et al., 2014; Diaz et al., 2018; Dramadri et al., 2019). Previous QTL studies based on GWAS have provided insight into the genetic architecture of yield in common bean. Kamfwa et al. (2015) identified significant SNPs for seed yield on Pv03 and Pv09, in agreement with the results found in the present study. More recently, Oladzad et al. (2019) identified SNPs associated with yield in a panel of Mesoamerican germplasm grown under heat stress in Honduras and Puerto Rico (*n* = 119 genotypes), placed on chromosomes 3, 8, and 11. The QTL identified on chromosome 3 was at the position 41,096,424 and explained 14% of the variation in yield, whereas in our study the QTL was placed at position 48,279,384 and explained 4% of the variance. Another QTL for GY identified by Oladzad et al. (2019) on chromosome 8 at 9,130 Mb was in a very close position to one of the six QTLs detected in our study (SNP 8207790 at 9,871 Mb, *r*^2^ = ∼6%). For the QTLs placed on chromosome 11, considering the three identified in our study, one was at similar position (SNP 8215509 at 49,353 Mb, *r*^2^ = ∼13%) to that previously identified by Oladzad et al. (2019) and placed at 47,305 Mb.

The detection of QTL affecting yield, under drought, would be of great interest for the design of molecular tools for marker-assisted selection and identification of target genes for edition, mainly when complemented by fine mapping studies (Tura et al., 2020). Despite a smaller number of QTLs identified under drought (10) compared to the irrigated condition (25), novel genomic regions were detected. Many of the morphological and physiological mechanisms linked to drought tolerance in common beans are known (Rosales et al., 2012; Polania et al., 2016; Lanna et al., 2018); however, to obtain an accurate phenotyping, adding to the large number of genes involved in the control of these traits (several of small effect) makes the drought tolerance at the genetic level difficult to be understood (Würschum, 2012). In our study, all identified QTLs (10) were detected in joint multienvironmental analyses, and most had a negative additive effect associated to the reference allele in the single-model GWAS, meaning that plants with the alternative allele increased trait performance. Only one QTL identified by multiexperimental joint analysis (7.93%) was also identified in individual environmental analysis (S01_1572065), which showed a high phenotypic contribution rate (11.56%). Wang et al. (2019) suggested that both the QTL additive effect and QTL × environment interaction act on reducing the phenotypic variation in the multienvironmental joint analysis, since the individual environmental analysis method only estimates the additive effects of QTL, which also seems to apply in the current study. In addition to that, all QTLs showed stability across the years of experimentation, and heritability ranged from 0.37 to 0.57 (moderate to high), indicating that the selection is likely to provide a benefit for common bean breeding, and the markers could provide promising targets for application in future studies. The higher proportion of phenotypic variations (17%) was explained by SNP 3383887 on chromosome 10, placed in a region comprising 4 SNPs, and SNP S01_1572065 (12%) placed on chromosome 1. The significant SNP 3383887 was located in gene Phvul.010G141600, of unknown function in common beans, encoding a protein homologous (74%) to the soybean transcription factor (C2H2 zinc finger), which was found to play an important role in abiotic plant stress (Guoliang et al., 2020), and SNP S01_1572065 located in gene Phvul.001G019600, which was annotated as a posttranscriptional gene regulator (polyadenylate-binding protein) that has an important biological function in plant adaptation to changing environments, in particular drought stress (Marondedze et al., 2019).

## Conclusion

This study reports the use of high-density genotypic data in a panel composed of 339 diverse Mesoamerican common bean accessions structured into Brazilian landraces and lines/cultivars from diverse origins (national and international germplasms). A large set of markers (11,870) based on DArTseq and CaptureSeq was successfully genotyped. The overall extension of LD decay along the genome, which is a relevant parameter in association studies between variants and traits, was estimated to be ∼63 kb, showing great variation in function of the evaluated germplasm (98–124 kb). In addition, important genomic regions under selection during landraces and lines/cultivars domestication were identified. The Mesoamerican germplasm panel was evaluated in field experiments conducted in 3 consecutive years in the presence and absence of water deficit and allowed the identification of genotypes with better performance for SW and GY promising to common bean breeding programs. A total of 189 QTLs were found associated with SW and 33 with GY, and many were within or near genes that have been reported to play important roles in biochemical and physiological processes related to GY, thus providing important clues on the mechanism of productivity under water deficit. These findings will be of great use for MAS of common bean varieties with improved SW and GY. This study makes available, under seed request, a panel of genotyped germplasm useful for GWASs of any relevant trait in common bean.

## Data Availability Statement

The datasets generated for this study can be found in Figshare, https://doi.org/10.6084/m9.figshare.12921437.

## Author Contributions

CG and RV conceived, designed, and conducted the field experiments. PV, LN, and RV performed the laboratory experiments. PV, BM, JA, OM, TB, IS, and AC analyzed the data. PV and RV wrote the manuscript. PV, AC, BM, OM, JA, MZ, LN, and CB revised the manuscript. All the authors contributed to the article and approved the submitted version.

## Conflict of Interest

The authors declare that the research was conducted in the absence of any commercial or financial relationships that could be construed as a potential conflict of interest.
