# Supplementary figures and images for: Genome-Wide Association Studies Detect Multiple QTLs for Productivity in Mesoamerican Diversity Panel of Common Bean Under Drought Stress
Source: Front Plant Sci. 2020 Nov 12;11:574674. doi: 10.3389/fpls.2020.574674 (PMC7738703; doi:10.3389/fpls.2020.574674)

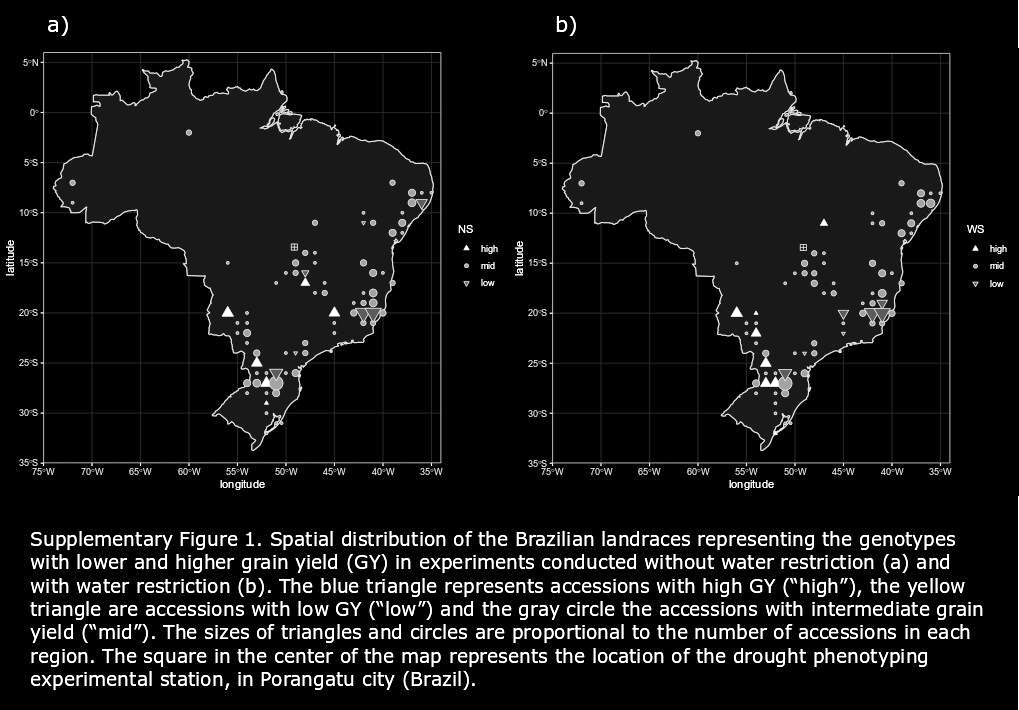

Supplement: Supplementary file 1 [file Image_1.tif]
