# Supplementary material for: Genome-Wide Association Studies Detect Multiple QTLs for Productivity in Mesoamerican Diversity Panel of Common Bean Under Drought Stress
Source: Front Plant Sci. 2020 Nov 12;11:574674. doi: 10.3389/fpls.2020.574674 (PMC7738703; doi:10.3389/fpls.2020.574674)
Supplement: Supplementary file 4 [file Table_2.docx]

**Supplementary Table 2** Temperature, relative humidity and rainfall in Porangatu (Goiás, Brazil), with maximum, minimum and average values, taken from the National Aeronautics and Space Administration (NASA*), for the years of experiments (2014, 2015 and 2016).

|  | **Maximum temp. (°C)** | **Minimum temp. (°C)** | **Relative humidity (%)** | **Rainfall (mm)** |
| --- | --- | --- | --- | --- |
|  | **2014** | | | |
| **minimum** | 26.9 | 16.5 | 26.9 | 0 |
| **maximum** | 39.9 | 26.5 | 78.9 | 9.77 |
| **average** | 34.2 | 20.5 | 49.5 | 0.21 |
|  | **2015** | | | |
| **minimum** | 28.0 | 18.21 | 23.6 | 0 |
| **maximum** | 40.2 | 26.17 | 85.7 | 6.1 |
| **average** | 34.7 | 21.0 | 46.3 | 0.17 |
|  | **2016** | | | |
| **minimum** | 33.3 | 18.8 | 24.6 | 0 |
| **maximum** | 39.8 | 27.4 | 58.4 | 0.15 |
| **average** | 36.8 | 22.2 | 35.8 | 0.005 |

* Link to NASA data: <https://power.larc.nasa.gov>
